# The Interacting Effects of Ungulate Hoofprints and Predatory Native Ants on Metamorph Cane Toads in Tropical Australia

**DOI:** 10.1371/journal.pone.0079496

**Published:** 2013-11-08

**Authors:** Elisa Cabrera-Guzmán, Michael R. Crossland, Edna González-Bernal, Richard Shine

**Affiliations:** School of Biological Sciences A08, University of Sydney, Sydney, New South Wales, Australia; Natural History Museum of Denmark, Denmark

## Abstract

Many invasive species exploit the disturbed habitats created by human activities. Understanding the effects of habitat disturbance on invasion success, and how disturbance interacts with other factors (such as biotic resistance to the invaders from the native fauna) may suggest new ways to reduce invader viability. In tropical Australia, commercial livestock production can facilitate invasion by the cane toad (*Rhinella marina*), because hoofprints left by cattle and horses around waterbody margins provide distinctive (cool, moist) microhabitats; nevertheless the same microhabitat can inhibit the success of cane toads by increasing the risks of predation or drowning. Metamorph cane toads actively select hoofprints as retreat-sites to escape dangerous thermal and hydric conditions in the surrounding landscape. However, hoofprint geometry is important: in hoofprints with steep sides the young toads are more likely to be attacked by predatory ants (*Iridomyrmex reburrus*) and are more likely to drown following heavy rain. Thus, anthropogenic changes to the landscape interact with predation by native taxa to affect the ability of cane toads in this vulnerable life-history stage to thrive in the harsh abiotic conditions of tropical Australia.

## Introduction

Under the Darwinian paradigm that species are intricately adapted to the areas they inhabit, we might expect that few taxa would be able to invade regions that experience climatic conditions very different from those in their native range. Although resemblance between native and invaded ranges (in terms of both abiotic and biotic factors) can indeed predict the probability of establishment of a species outside its natural distribution [1], there are many examples where invaders have thrived under conditions that, at least at first sight, seem far removed from those to which they have adapted over evolutionary time. That success reflects genotypic and phenotypic plasticity, and broad environmental tolerances [2], [3]. For example, the green-and-golden bellfrog (*Litoria aurea*) has a natural distribution in temperate eastern Australia, but has been successfully translocated to very different climates in the oceanic islands of New Zealand, Vanuatu and New Caledonia [4], [5]. One key to this flexibility is reliance upon the distinctive habitats created by human activities. Even if the surrounding landscape contains many novel challenges, the areas immediately around human settlements may bear strong similarities between the invaded range and the natural distribution [6], [7]. Thus, many anthropogenically modified habitats are prone to invasion [8].

Habitat disturbance clearly facilitates invasion of commensal taxa such as black rats (*Rattus rattus*), house geckos (*Hemidactylus frenatus*), house sparrows (*Passer domesticus*) and Lesser Antillean whistling frogs (*Eleutherodactylus johnstonei*) [9], [10], [11], [12]. The same may well be true for a wider suite of invaders that rely on habitat disturbance wrought by human activities, but over a broader spatial scale (i.e. away from the immediate vicinity of buildings). In contrast, there are few documented cases in which habitat disturbance inhibits the success of invasive species [13]. To understand how habitat modifications might affect invader success, we can look in detail at the microhabitats created by disturbance, how those microhabitats are used by invaders, and the nature of advantages or disadvantages that result in these patterns of usage.

An important source of anthropogenic habitat disturbance is the trampling by large domestic herbivores. This can influence the physical, chemical and microbial properties of the soil, generating substantial variation in microtopography [14], [15]. Hoofprints left in the soft mud make a more heterogeneous environment for small fauna, turning relatively flat surfaces into rugose ones, and creating deep cavities that provide abiotic conditions not available in the undisturbed local habitat. Domestic herbivores can thus be considered as physical ecosystem engineers that modify, maintain or create habitats [16], [17]. For example, cattle hoofprints that retain water are used by mosquitoes as breeding habitat [18], [19] and such hoofprints can also reduce post-dispersal movements of seeds (and thus, rates of seed loss) on steep slopes [20]. Hence, the distribution and abundance of many animal species can be affected either positively or negatively by the activities of buffalo, cattle and pigs in Australian ecosystems [21], [22], [23], [24].

In the present paper, we explore the effects of commercial livestock production on aspects of habitat availability and use by invasive cane toads (*Rhinella marina*) in tropical Australia, and examine how livestock-induced habitat modifications have affected the interaction between invasive toads and native predators.

Specifically, we quantified domestic stock (cattle and horse) hoofprint characteristics, use of hoofprints as microhabitat by metamorph cane toads, and the extent to which hoofprint geometry affects vulnerability of toads to predation by native ants.

## Materials and Methods

### Ethics Statement

The field experiments performed in this study were conducted at one location: the margins of a permanent pond located on private land at Middle Point Village, Northern Territory, Australia (12°34′39″ S, 131°19′05″ E). This area does not belong to a National Park or other protected area of land. Permission for research at this location was granted by the owners of the property. The Parks and Wildlife Commission of the Northern Territory (permit numbers 32289 and 40463) and the University of Sydney Animal Ethics Committee (Protocol L04/6-2010/3/5333) approved the procedures in this project.

The study was executed with an invasive species (the cane toad: *Rhinella marina*) and an abundant ant species (the meat ant: *Iridomyrmex reburrus*) and did not involve endangered or protected species. For some field experiments, metamorph cane toads were collected by hand and kept in take-away plastic containers with some water for a few minutes before being tested. A small number of ants were collected and kept in large plastic containers for a few minutes to prevent their ingress into experimental field enclosures during trials.

### Study area

Our field site is located in the Adelaide River floodplain, 60 km east of Darwin, Northern Territory, in the Australian wet-dry tropics. The climate is hot year-round, with mean maximum monthly temperatures exceeding 30°C in every month of the year [25]. Precipitation is highly seasonal, however, with >75% of the annual rainfall recorded in less than four months per year (broadly, January through March, the “wet-season”): [25], [26]. Thus, the landscape dries out progressively from April through to November. Our study site lies within an area of fenced paddocks dominated by exotic pasture grasses and occasional trees. Most of this area is used for livestock production, such that horses (*Equus caballus*), cows (*Bos taurus* and *B. indicus*) and Asian water buffaloes (*Bubalus bubalis*) are common across the landscape. One of the most obvious effects of these ungulates (both perissodactyl and artiodactyl) is the creation of hoofprints in the wet mud.

We worked around the margins of a permanent pond located at Middle Point Village (12°34′39″ S, 131°19′05″ E), approximately circular with a maximum diameter of 40 m and a maximum depth of 1.5 m. The pond is surrounded by grass, but with bare muddy margins for most of the year. It is used by cane toads as a breeding site [27] mostly when the pool diameter averages 25 m and the edge is unvegetated. This area is also a major watering-point for domestic stock, which leave deep footprints in the mud along the waterbody margins.

Hoofprints vary in diameter, shape and depth as a function of soil type, soil water content, animal species, body size, gait, and time since formation [28], [29], [30]. Metamorph cane toads often can be found clustered within particular hoofprints (James 1994 unpublished data), suggesting that the microhabitat provided by these might be important for toads. Also, metamorph toads in this system are vulnerable to attack by predatory meat ants [27], [31], [32], whose activities plausibly might be either facilitated or hindered by the presence of hoofprints.

### Study species

The cane toad (*Rhinella marina*, formerly *Bufo marinus*) is a large and highly toxic bufonid anuran native to the Americas. Toads were brought to north-eastern Australia in 1935 to control insect pests of commercial agriculture, and have since spread rapidly across tropical Australia, causing significant negative impacts on native animals [33], [34], [35]. In our study area, cane toads typically breed when ponds are drying during the mid-year months, or late in the year when temperatures are high and precipitation is minimal [32]. Like most anurans, cane toads desiccate rapidly if exposed to dry air and hence, adult toads select cool moist retreat-sites during the heat of the day (e.g. crevices between rocks, burrows, hollows under trees, leaf litter, grass and other dense vegetation: [36], [37], [38]). Recently metamorphosed toads are even more vulnerable to environmental extremes, both because of their small body sizes (and thus, high ratios of surface area to volume compared to adults: [39], [40]), and because they are active diurnally (apparently to avoid being consumed by larger conspecifics: [41]). The dangers of overheating and desiccation restrict newly-metamorphosed toads to the margins of the natal pond [42], [43], [44], [45]. Densities of metamorph toads can exceed >100 per m^2^ around water bodies in this area [31]. Because cane toads spawn primarily in ponds with open (unvegetated) ground margins, gently sloping banks, and anthropogenic disturbance [46], [47], sheltered (moist, cool, safe) retreat-sites close to the water's edge can be rare (E. Cabrera-Guzmán unpublished data).

The margins of these waterbodies also contain high densities of foraging ants, notably “meat ants” (Dolichoderine – *Iridomyrmex purpureus* and allies). These species are dominant members of Australian ant communities because of their abundance, biomass, high activity rates and aggressiveness [48], [49]. They overlap widely with cane toads in Australia in terms of distributional range, diel activity cycles and preferred habitats, with the species *Iridomyrmex reburrus* being a major predator on post-metamorphic toads at our study site [27], [31], [32]. Unlike many vertebrate predators, these ants are not affected by the toad's toxins [50]. During our field observations and experiments (July to November, 2009 and 2011, and July to December, 2012), our study pond was surrounded by 36 active meat ant nest entrances (holes). These ants set up highly organized foraging trails to transport resources to their nest [51].

### Densities and sizes of hoofprints relative to distance from the pond edge

To quantify the numbers and characteristics of hoofprints around toad breeding ponds, we surveyed three ponds (the study pond plus two temporary ponds located in and near Middle Point Village). We set out a series of quadrats (each 1 m^2^) along linear transects (each 6 m long and at least 1 m apart, beginning at the pond edge and extending perpendicularly away from the pond). We recorded the number of complete hoofprints inside each quadrat, and the distance of the quadrat from the water's edge. We measured the maximum depth and diameter of each of these hoofprints. Measurements were taken during the dry season when hoofprints near the water's edge retained humidity whereas hoofprints away from the water's edge were dry.

### Abundance and use of microhabitats by metamorph cane toads

We scored the number of live metamorph toads and the microhabitats in which they were found during the dry season, along 10 linear transects (each 7 m long, at 8-m intervals on the perimeter of the study pond and extending perpendicularly away from the pond edge). On each transect we counted metamorph toads in 50×50 cm quadrats (previously marked on the ground surface), separated by 30 cm. Counts were made at three times of day (0800–1000 h, 1200–1400 h, 1600–1800 h) over nine days.

### Thermal and hydric regimes in hoofprints and adjacent pond margins

To quantify the abiotic conditions available to metamorph cane toads during the dry season, we deployed thermocron temperature loggers (iButton®, USA) inside 17 hoofprints (randomly selected within three metres of the pond edge) and on 17 adjacent open substrates next to these prints, for a seven-day period. Thermocrons were programmed to record temperature at 1-h intervals. To measure likely rates of desiccation of metamorph toads in each of these two microhabitat types, we used 2% agar models (which accurately simulate rates of water loss by toads: see [36], [43]). A total of 120 agar models (similar in mass and physical dimensions to metamorph toads) were placed on open substrates (60) and in hoofprints (60). Models were 7 mm^2^ square, and weighed an average of 0.41 g (A&D Company Limited digital balance, FX-200*i*WP, USA). We set out pre-weighed agar models for five-hour periods (0700–1200 h; 1300–1800 h) before collecting and reweighing them to calculate desiccation rates as mass loss.

### Does the shape of a hoofprint influence its use by toads?

Our surveys revealed significant variation in hoofprint attributes. A horse or cow walking down a steep muddy slope produces relatively shallow hoofprints, with a long sloping edge where the hoof has slid down through the mud. In contrast, a horse or cow stepping onto the flat muddy pond edge sinks in with little slippage, leaving a hoofprint with steep sides. We defined these two types of hoofprints as “gently-sloping” and “steep-sided” respectively. Plausibly, they may differ in their usefulness to metamorph toads: for example, the steep-sided hoofprints can be more difficult for a toad to escape from. To clarify the responses of toads to these two types of hoofprints, we set up four enclosures (80×60 cm; 30 cm high) near the edge of the pond, constructed of fibrocement sheets on three sides and a glass sheet on the other (to prevent the formation of a patch of shade). The walls of the enclosures and manual collection of ants, when necessary, prevented their ingress to the inside area. Half the enclosures contained two artificially created gently-sloping hoofprints while the other half contained two artificially created steep-sided hoofprints. We created these artificial hoofprints in the middle of each enclosure using a horseshoe nailed to a stick (plus excavation). The dimensions of our standardised artificial hoofprints were based on our measurements of 127 natural hoofprints made by horses and cattle in the mud (within 2 m from the water's edge) around water bodies inhabited by toads. Based on mean values of those measurements, all artificial hoofprints were 11.5 cm in diameter, with a maximum depth of 7.8 cm; in the gently-sloping hoofprints, the sloping portion comprised 34% of the perimeter. We started trials one day later, by spraying some water in the enclosures and the hoofprints to homogenize conditions, and then adding five metamorph cane toads (randomly selected from the edge of the study pond; mean snout-urostyle length (SUL): 10.99 mm, range: 9.07–12.6 mm; mean mass: 0.151 g, range: 0.091–0.21 g). The toads were placed in a corner of the enclosure, and then observed for 30 min to determine how many entered the hoofprints (which represented 8% of the total surface of the enclosures). All trials (N = 14 and 15 per type of print) were performed during the dry season under sunny conditions, between 0900 and 1800 h, and each toad was used in only a single trial.

### Toad use of gently-sloping hoofprints versus other types of microhabitats

To clarify toad selection of hoofprints compared to other types of microhabitats during dry conditions, we used field enclosures (as described above) near the edge of the pond. As well as two artificial gently-sloping hoofprints (made as in the previous experiment), each enclosure contained two large water-lily leaves with similar surface area to that of the hoofprints (mean leaf length: 12.78 cm, range: 9.5–16 cm; mean diameter: 12.77 cm, range: 10–15 cm; mean hoofprint surface length: 15 cm, range: 13–18 cm; mean diameter: 12.66 cm, range: 12–14 cm). We also kept or created 20 open cracks (mean length: 12.25 cm, range: 4.0–18.3 cm; mean width: 0.58 cm, range: 0.5–0.8 cm) in each enclosure. Our two experimental treatments were: (1) 10 metamorphs in an enclosure with two hoofprints, two leaves, and 20 cracks (28 replicates), and (2) 10 metamorphs in an enclosure with no hoofprints, two leaves and 20 cracks (14 replicates). For each trial, we released 10 metamorph toads (mean SUL: 10.84 mm, range: 10.05–11.75 mm; mean mass: 0.14 g, range: 0.11–0.16 g) into each enclosure at 1900 h, when meat ant activity decreased (see [32]). We checked the location of the animals at 0700–0730 h the next morning. We repeated these trials with a new group of 10 metamorphs at 1000 h, and checked toad locations at 1400 h.

### Effects of ants on the use of hoofprints and survival rates of metamorph cane toads

To evaluate the idea that ants may affect toads differently in hoofprints compared to open substrates, we created flat open areas at the muddy edge of the waterbody with the two types of artificial hoofprints as explained for previous experiments. We marked a 30 cm^2^ quadrat centred on each hoofprint, and another 30 cm^2^ quadrat on the adjacent open (flat) substratum. The following day, we placed groups of five metamorph toads either on open substrates, in an artificial gently-sloping hoofprint, or in an artificial steep-sided hoofprint. Orthogonal to these treatments, we excluded predatory ants from half of the quadrats with fibre-cement sheets (as described above) to create an enclosure 85×80 cm, 30 cm high. The experimental toads (mean SUL: 10.63 mm, range: 10.7–12.01 mm; mean mass: 0.14 g, range: 0.12–0.17 g) were collected on the edges of the pond, with each toad being used in only a single trial; we ran 16 to 19 replicates per treatment. Trials were performed during both dry and wet seasons.

For each trial we placed the metamorph toads in the centre of the quadrat (if appropriate, inside the hoofprint). Beginning one minute later, we observed the experimental animals for 30 min to document the time that each metamorph spent in the quadrat. For treatments where ants were present, we also recorded the total number of ants arriving, the number of attacks on metamorphs (bites or grabs), the number of times that a metamorph moved away from an approaching ant (avoidance), and the number of successful predation events (death of toads).

Finally, during both dry and wet seasons, we performed a habitat-manipulation experiment to quantify mortality rates of metamorph toads in gently-sloping versus steep-sided hoofprints. We modified the structure of 54 natural hoofprints around the edge of the study pond (all within 1 m of the water's edge) to create 27 groups of three possible microhabitats: one standardised gently-sloping hoofprint, one steep-sided hoofprint, and a patch of flat open substrate (all within a 20 cm^2^ quadrat). We left the area to be occupied by metamorph toads and meat ants, and recorded the number of live and dead (presumably ant-killed) toads in each of the three types of microhabitats at three times of day (0800 h, 1400 h, 1800 h) over seven days. Preliminary observations showed that any toads killed by ants were removed within four hours, so it is unlikely that any of the dead toads were counted more than once.

### Other mortality sources associated with hoofprints

After heavy rains during the wet season, hoofprints sometimes contained pools of water, and small toads (especially metamorphosing individuals) drowned within those pools, likely because they were unable to climb the steep walls of the hoofprint. To quantify this phenomenon, we counted drowned toads within a random subset (26 of 54) of the modified hoofprints used in the previous experiment, on three successive days when rain fell heavily in the early afternoon. The counts (one per day, over a 3-day period) were conducted immediately after the rain had finished each day (between 1600 and 1700 h).

### Data analysis

We used ANOVA and post hoc Tukey tests to compare the number of hoofprints, their depth and diameter at different distances from the water's edge around ponds. The numbers of toads in each of the pre-defined microhabitat types were compared among the three time periods using repeated measures ANOVA with habitat type as the repeated measure, and time of day as the factor. We compared the numbers of toads entering the two types of hoofprints (gently-sloping vs steep-sided) in our enclosures using contingency-table analyses. Mean temperatures and desiccation rates (the latter based upon proportion of mass loss of the agar models) were compared between hoofprints and the adjacent open substrate using t-tests.

In the trials where we provided an array of potential microhabitats, we evaluated if the presence of hoofprints modified (1) the proportion of toads in the quadrat that used the other types of microhabitats; and (2) the toads' relative usage of each of those other types of microhabitats (i.e. omitting toads inside hoofprints). We used repeated measures ANOVA with presence of hoofprints, time of day and the interaction presence of hoofprint*time of day as factors. The repeated measure was the number of toads in each microhabitat type. We evaluated whether the presence of hoofprints or time of day modified the relative numbers of toads in the open *versus* under shelter objects, and also tested microhabitat preference with ANOVA and posthoc Tukey tests.

To evaluate the response of metamorph toads to meat ants, we compared the mean duration of toad residence inside the marked quadrats with open substrates *versus* gently-sloping hoofprints *versus* steep-sided hoofprints. The time spent in each quadrat by the five individual toads in each replicate trial was averaged, to yield a single value per trial (thus avoiding pseudoreplication). For treatments with ants present, we used ANOVA and ANCOVA to compare treatments with respect to the total number of ants arriving, the number of ant attacks on metamorphs, the number of times that toads avoided ants, and the number of successful predation events (dead toads). We also used ANOVA to compare the numbers of live and dead cane toads (killed by meat ants) among the three kinds of microhabitats, using two factors (time of day and type of microhabitat). The numbers of drowned metamorphs were compared between sloping hoofprints and steep-sided hoofprints using t-tests. All analyses were performed using JMP 5.0.1 software [52] and VassarStats [53].

## Results

### Densities and sizes of hoofprints relative to distance from the pond edge

**Figure 1:**
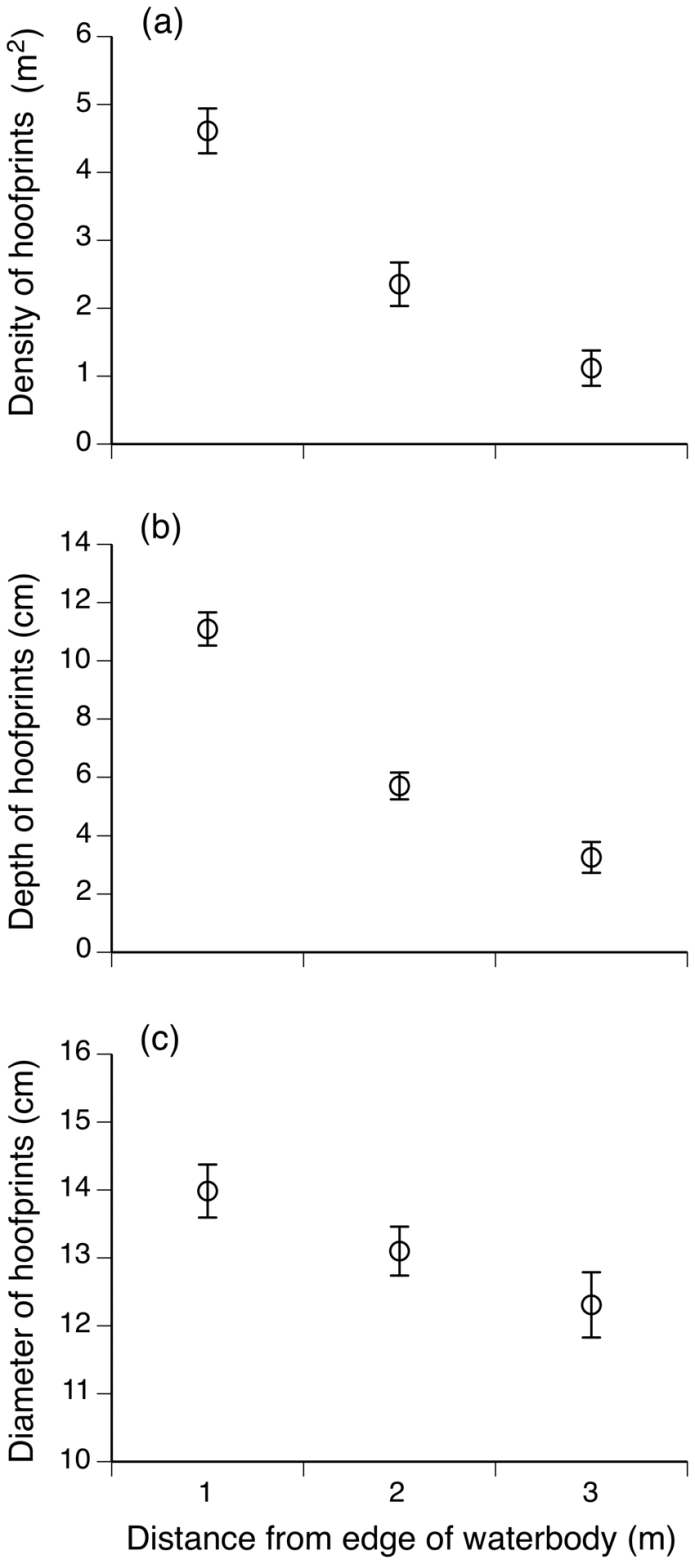
Characteristics of hoofprints made by domestic ungulates (cattle, buffaloes, and horses) at different distances from the edge of three waterbodies in tropical Australia. The panels show means ±1 SE of density (a), depth (b), and diameter (c) of the hoofprints that we measured.

Hoofprints were very common at the studied ponds, with an average density of 4.6 per m^2^ in the first meter from the water's edge (Figure 1a). The mean number of hoofprints per quadrat decreased with distance from the water (*F_2,41_* = 20.28, *P*<0.0001; there were significantly more hoofprints at 1 m from the edge than at 2 or 3 m: Tukey *P*<0.05). We did not find any hoofprints farther than 3 m distance from the edge of the pond (Figure 1a). Hoofprints closer to the water's edge also were deeper and wider than those at 2 or 3 m away from the edge (depth: *F_2,41_* = 52.60, *P*<0.0001, 1>2 = 3 m, Tukey *P*<0.05; diameter: *F_2,41_* = 3.75, *P* = 0.031; 1>3 m, Tukey *P*<0.05; Figure 1b, c).

### Abundance and use of microhabitats by metamorph cane toads

**Figure 2:**
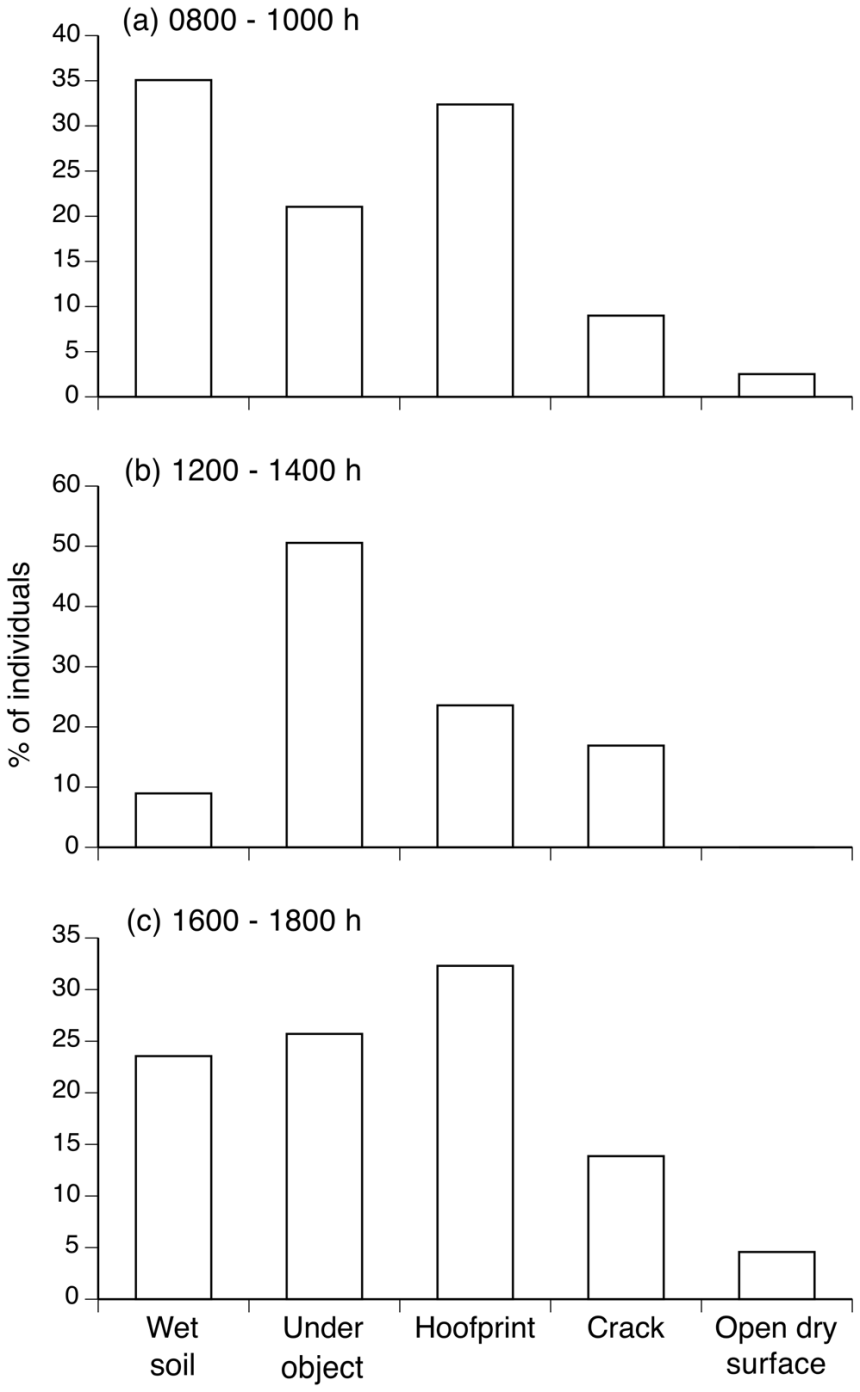
Number of metamorph cane toads using different microhabitats in the field. Surveys were conducted in the morning (a), around midday (b), and in the afternoon (c). “Under object” includes spaces under leaf litter, leaves, plants, logs, branches, pieces of dry mud, and rocks. “Open dry surface” includes dry soil, leaf litter and grass.

Our surveys around the pond's edge showed that metamorph cane toads often were found on flat open substrates, but also sheltered under objects, or inside hoofprints or soil cracks (Figure 2a, b, c). The relative numbers of toads in the five recognized microhabitat types did not change significantly through the course of the day (*F_2,27_* = 0.10, *P* = 0.91). However, few metamorphs were found on open wet soil and none was found on open dry locations exposed to direct sunlight at midday (Figure 2b).

### Thermal and hydric regimes in hoofprints and adjacent pond margins

Conditions inside the hoofprints were cooler than on the adjacent open substratum (respectively: mean 24.04±0.08, range 14.5–37.5°C *versus* mean 26.62±0.22, range 11.0–58.5°C; *t* = 10.89, *df* = 5186, *P*<0.0001). Agar models in hoofprints also desiccated less quickly than did those on open ground (mean % loss 50.01±2.36 vs 72.32±2.23; *t* = 6.82, *df* = 112, *P*<0.0001).

### Does the shape of a hoofprint influence its use by toads?

During our 30-min trials, 42 of the 70 metamorph toads (60%) entered gently-sloping hoofprints, while only 11 of 75 (15%) entered steep-sided hoofprints; this difference was significant (chi-square = 32.08, *df* = 1, *P*<0.0001).

### Toad use of gently-sloping hoofprints versus other types of microhabitats

**Figure 3:**
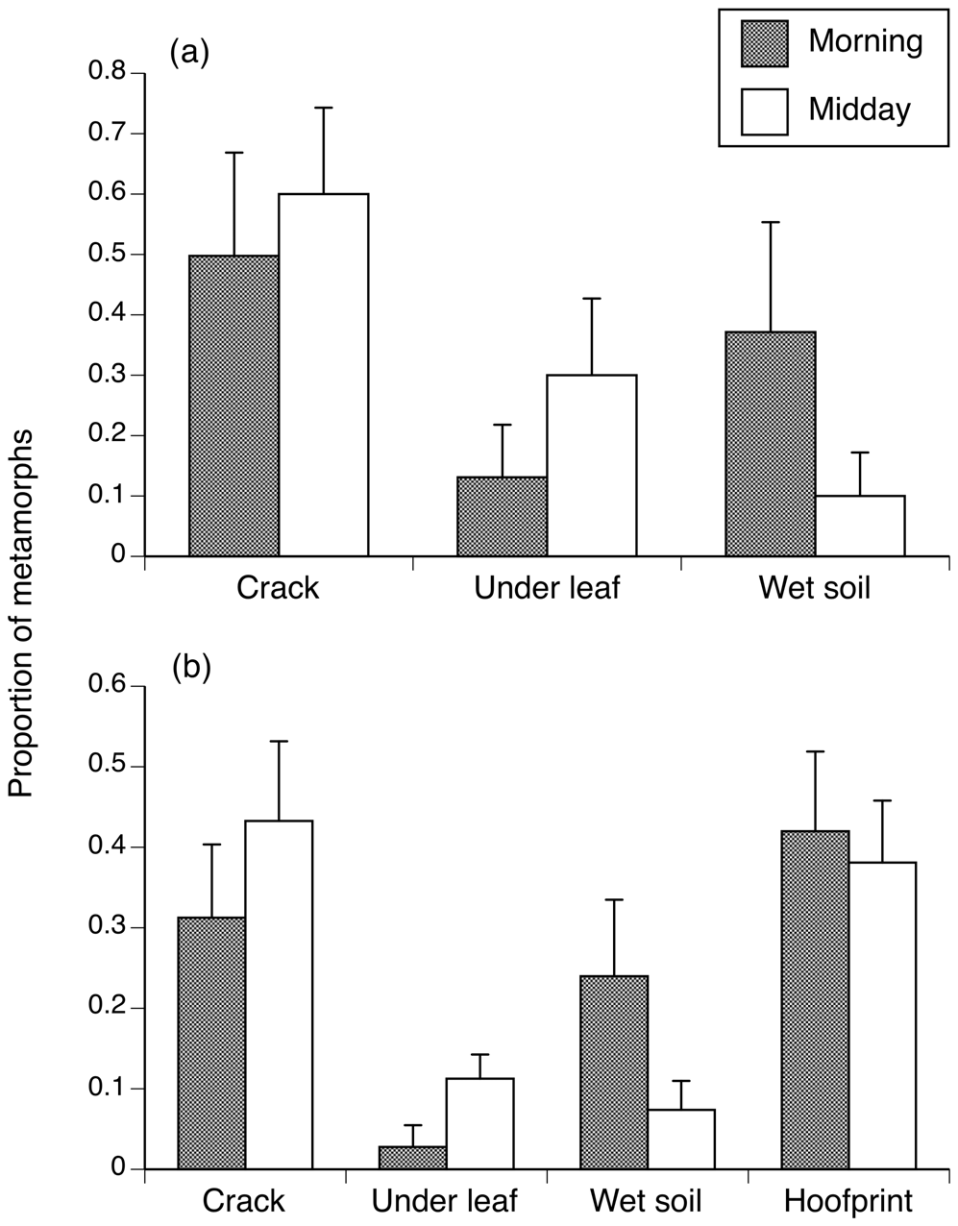
Proportion of metamorph cane toads (mean +1 SE) using different microhabitats in field enclosures. The panels show proportions with no hoofprints (a) and with hoofprints present (b). Dark bars show data collected during morning surveys, whereas open bars show data from midday surveys.

Unsurprisingly, adding hoofprints to an enclosure changed microhabitat use by toads (*F_1,35_* = 20.28, *P*<0.0001), with 40% of the toads using hoofprints when these were available. The analysis did not reveal any other significant effects on microhabitat use (time of day: *F_1,35_* = 0.05, *P* = 0.83; presence of hoofprints*time of day: *F_1,35_* = 0.05, *P* = 0.83). When leaves, soil cracks and wet soil were the only available microhabitats, metamorph toads selected soil cracks over leaves (*F_2,36_* = 3.99, *P* = 0.02; Tukey *P*<0.05; the use of wet soil did not differ from the other two microhabitats, Tukey *P*>0.05; Figure 3a). When hoofprints were available, the metamorphs preferred hoofprints and soil cracks over leaves or wet soil (*F_3,100_* = 9.39, *P*<0.0001; Tukey *P*<0.05; Figure 3b).

### Effects of ants on the use of hoofprints and survival rates of metamorph cane toads

**Figure 4:**
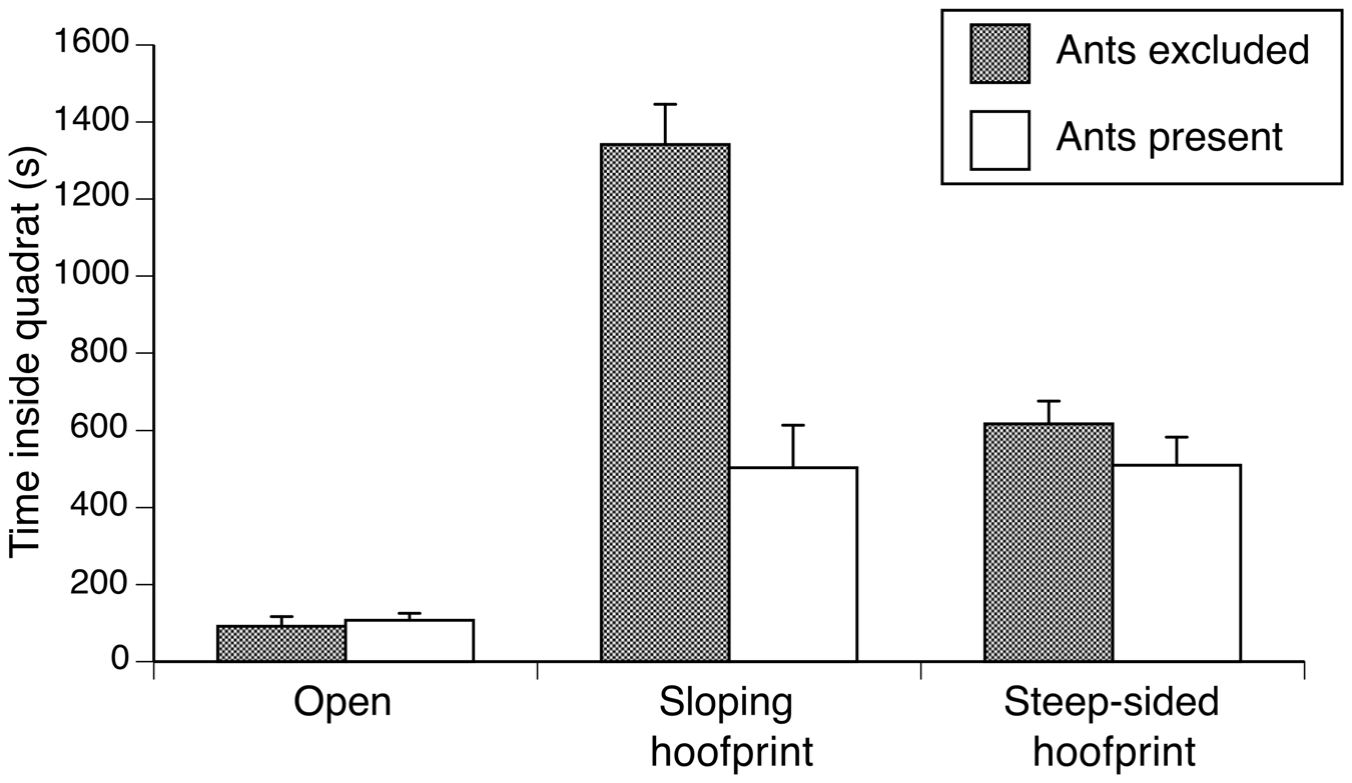
Mean time (+1 SE) spent by metamorph cane toads in quadrats with open substrate, a gently-sloping hoofprint or a steep-sided hoofprint in the presence vs absence of foraging meat ants.

When ants were excluded, metamorph toads stayed for longer periods of time in quadrats with hoofprints than in open quadrats (*F_2,48_* = 77.18; *P*<0.001; open < steep-sided < gently-sloping, Tukey *P*<0.05). When predatory ants were present, the toads stayed longer in quadrats with both kinds of hoofprints than on open substrate (*F_2,49_* = 9.00; *P* = 0.0005; Tukey *P*<0.05) but spent significantly less time in sloping hoofprints (*F_5,97_* = 38.88; *P*<0.0001, Tukey *P*<0.05; Figure 4).

**Figure 5:**
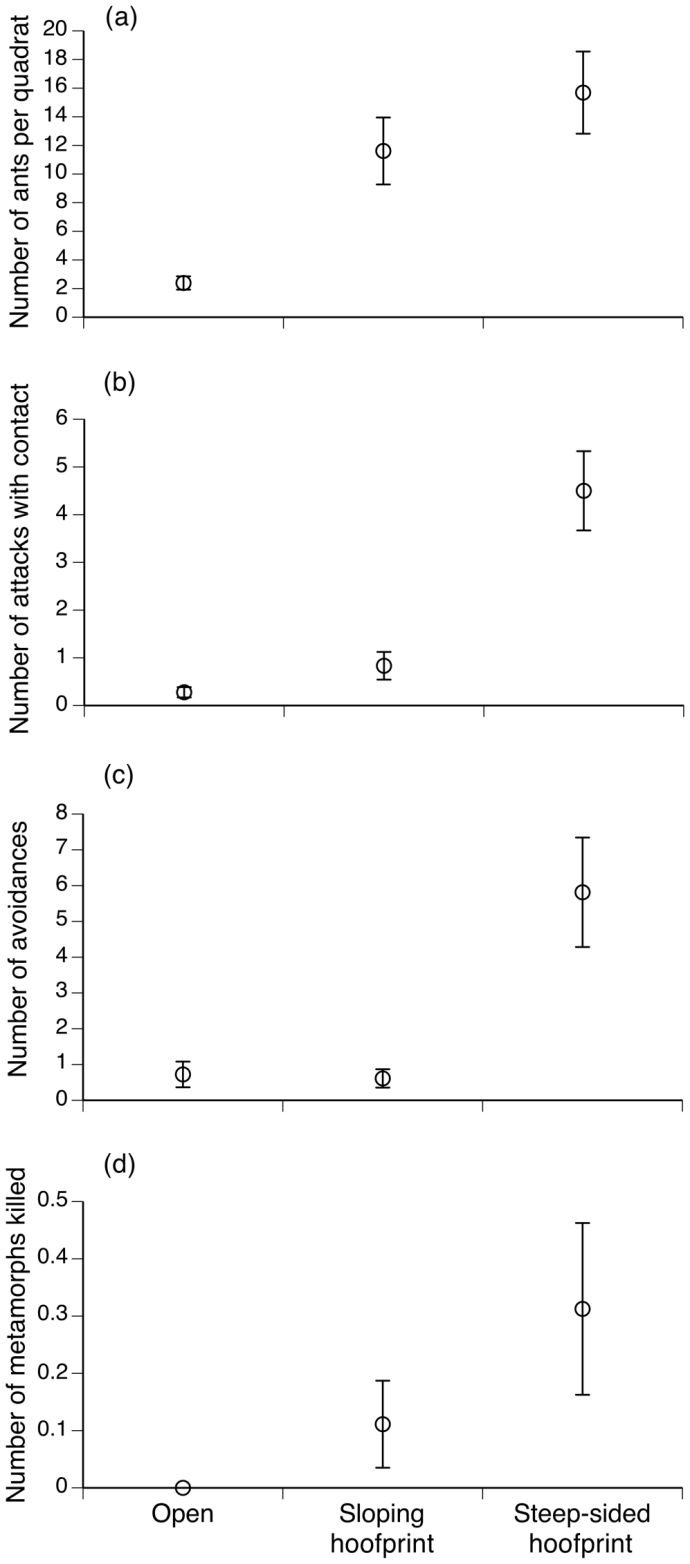
Vulnerability of metamorph cane toads to predation by ants on open substrates and in hoofprints. The panels show the effects of microhabitat on the number of ants per quadrat (a), the number of times that toads were attacked (seized or bitten) by ants (b), the number of times that toads avoided ant attack by moving away (c), and the number of metamorph toads killed by ant attack (d). Data are means ±1 SE.

In treatments that allowed access by ants, quadrats with hoofprints (of both types) attracted more ants than did those lacking hoofprints (*F_2,49_* = 9.99; *P* = 0.0002; Tukey *P*<0.05, Figure 5a). The number of times that metamorph toads were attacked by ants, and the number of times that toads moved to avoid ant attacks, were higher in steep-sided hoofprints than on open substrates or in gently-sloping hoofprints (contacts: *F_2,49_* = 21.87, *P*<0.0001, Tukey *P*<0.05; escapes: *F_2,49_* = 11.51, *P*<0.0001, Tukey *P*<0.05; Figure 5b, c). No successful predation events were recorded on open substrates, whereas many toads were killed in steep-sided hoofprints (Figure 5d). High variances meant that the number of successful predation events did not differ significantly among shelter types (*F_2,49_* = 2.86, *P* = 0.07) unless we used a one-tailed test based on the *a priori* prediction of higher risk to toads in steep-sided footprints (so, *P*<0.04).

ANCOVA with ant densities as a covariate showed that the higher rate of attacks by ants in steep-sided hoofprints was not a simple result of higher densities of ants in those hoofprints (shelter type: *F_2,46_* = 8.99, *P* = 0.0005; total number of ants: *F_1,46_* = 0.16, *P* = 0.69; shelter type*total number of ants: *F_2,46_* = 0.10, *P* = 0.91). The number of ant-avoidance behaviours by toads increased at higher ant densities (*F_1,48_* = 8.31, *P* = 0.006) but at the same ant densities, the number of avoidance responses was higher in steep-sided hoofprints than in the other two microhabitat types (*F_2,48_* = 7.15, *P* = 0.002; microhabitat type*ant density: *F_2,46_* = 2.897, *P* = 0.06).

**Figure 6:**
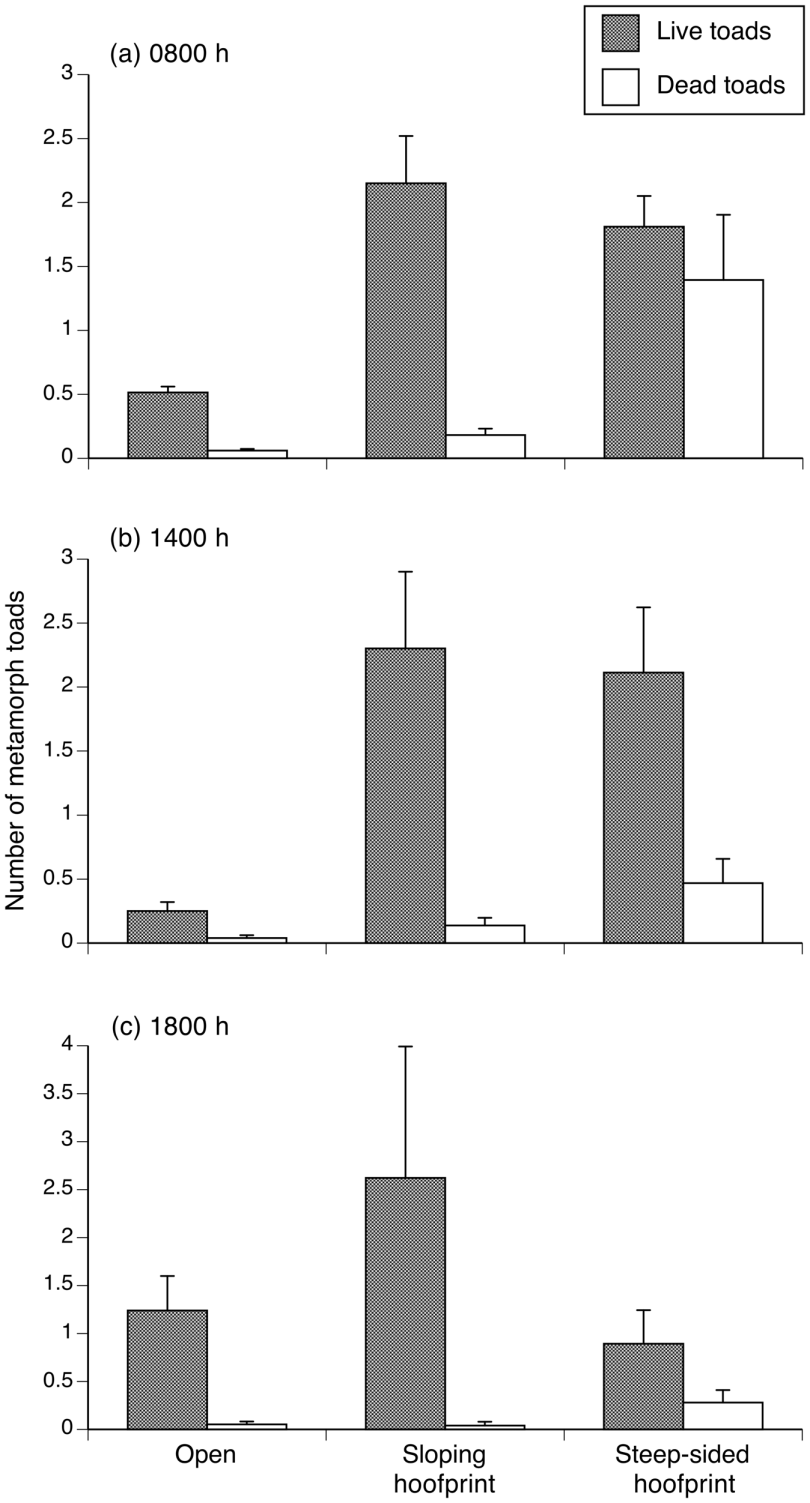
Number of toads (mean +1 SE) found alive and dead (killed by meat ants) on open substrate and in hoofprints, during our field surveys. The panels show counts made in the morning (a), in the early afternoon (b), and in the late afternoon (c).

Our surveys of the relative numbers of live and ant-killed toads showed a significant interaction between time of day and substrate type (*F_4,898_* = 4.73, *P* = 0.009). That interaction term complicates interpretation, so we restricted the comparison to only steep-sided *versus* gently-sloping hoofprints. The number of dead toads relative to live toads was higher in steep-sided hoofprints than in gently-sloping hoofprints (alive/dead*hoofprint type: *F_1,549_* = 14.42, *P* = 0.0002; see Figure 6).

### Other mortality sources associated with hoofprints

**Figure 7:**
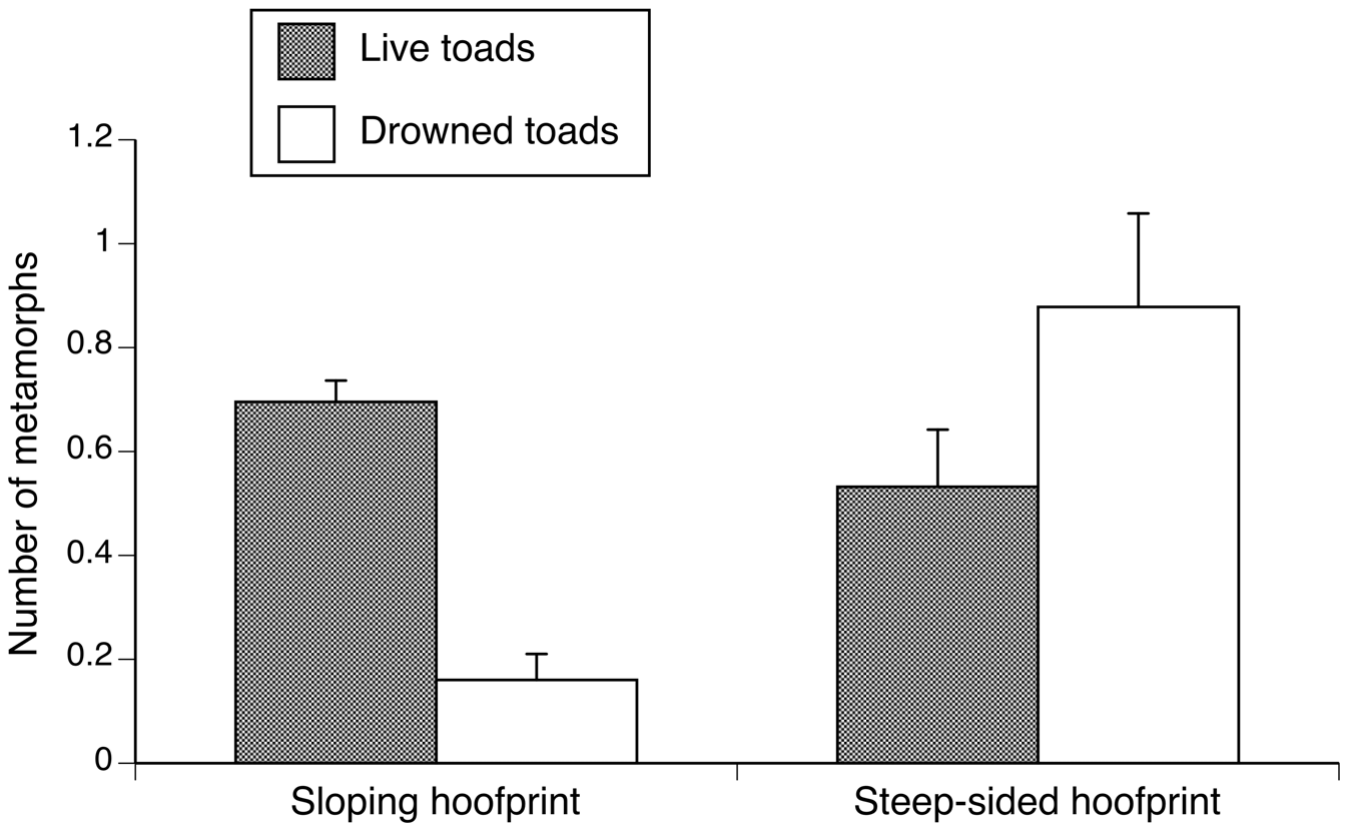
Number of toads found alive and dead (drowned) in hoofprints, during our field surveys performed immediately after a rain had finished.

Surveys after heavy rain showed that both kinds of hoofprint accumulate water, but differ in the risk that this poses to young toads; metamorphs can escape better from hoofprints with a gently-sloping side but not from a steep-sided hoofprint. Thus, the numbers of drowned toads were higher in steep-sided hoofprints than in gently-sloping hoofprints (*t* = 3.71, *df* = 41, *P* = 0.0006; Figure 7).

## Discussion

The drying muddy edges of ponds in agricultural landscapes in tropical Australia are pock-marked with the hoofprints of domestic livestock that use the ponds as watering sources. The large native mammals of Australia are all marsupials, with soft-padded feet that do not create deep depressions with sharp outlines, even in soft mud [29], [54]. Large wading birds such as black-necked storks (jabirus) foraging at the edge or in the shallows in the water bodies [55] sometimes create footprints that persist in drying mud, but these are smaller and shallower than those produced by horses and cattle. Previous studies have shown that commercial livestock production enhances habitat quality for cane toads by providing water sources in dry areas [56], and by providing cowpats that toads can use to rehydrate [57]. The present study suggests that livestock also transform the physical characteristics of waterbody margins in ways that are important to metamorph toads during the long tropical dry-season [40], [42]. Those changes can either improve habitat quality for toads, or decrease it. Because most anurans (including cane toads) experience high rates of water loss during warm and dry conditions [58], the risk of desiccation and overheating restricts metamorphs to pondside habitats for several months, increasing competition for food, and reducing survival rates through cannibalism and predation by ants [41], [43], [45], [59]. Our studies confirm that hoofprints provide more favourable thermal and hydric regimes than those available on the ground surface, and that metamorph toads actively select hoofprints (and indeed, particular types of hoofprints: ones with gently-sloping sides) as shelter sites. However, toad usage of this kind of hoofprint is reduced by predatory ants: the ants are drawn to hoofprints, and tend to drive toads away from these preferred shelter-sites. In contrast, toads do not enter steep-sided hoofprints as frequently as gently-sloping hoofprints, usually stopping at the edge of a steep-sided hoofprint and then changing their direction. Steep-sided hoofprints expose metamorphs to higher rates of attack by meat ants, in an enclosed space where escape is more difficult.

In our study system, both hydric and thermal benefits are likely to accrue to metamorphic anurans that use hoofprints as diurnal shelter-sites. Metamorphs that can move further away from the pond edge during relatively cool times of day benefit from higher food abundance, but their ability to do so is constrained by the dangers of desiccation and overheating [43]. Hoofprints provide moist cool refuges away from the water, facilitating access to food resources not available along the crowded pond edge. These hoofprints can be long-lasting (when no disturbance or rainfall are present); for example, metamorph toads continued to use two artificial gently-sloping hoofprints (7 m away from the edge of the pond) several months after we performed our experiments. Prey availability likely is enhanced by hoofprint structure also, with small (edible) mites, ants, beetles and other insects either trapped within them (i.e. after falling into steep-sided hoofprints) or attracted to the favourable abiotic conditions that are available within hoofprints. Some of these arthropods are preyed upon by metamorph toads (E. Cabrera-Guzmán unpublished data).

More generally, high densities of animals (including toads as well as insects) inside hoofprints plausibly could be attributed either to active selection of a refuge that provided protection from environmental extremes, or to passive capture of animals that fell into the hoofprint and were unable to escape due to its steep sides. Our experiments strongly suggest that metamorph toads actively exploit sloping hoofprints. Many toads entered gently-sloping hoofprints in enclosures with ants excluded, and the toads rapidly left such hoofprints when predatory ants arrived. These patterns are not consistent with the idea that hoofprints contain toads because the animals fall in, and find it difficult to leave. At least in the case of gently-sloping hoofprints, high numbers of metamorph toads in these sites likely are the result of active habitat selection. We suspect that the same is true for other anuran species that use these hoofprints as shelter sites, including small frogs such as *Crinia bilingua* and *Litoria microbelos* in our own study area. In contrast, the low numbers of toads entering steep-sided hoofprints in enclosures suggests that they may act as traps rather than as favoured habitat patches. Under natural field conditions (i.e. outside enclosures) we observed some toads falling into steep-sided footprints while escaping from attack by predatory ants, or after being alarmed by the movement of a large animal (e.g. ungulates, birds). Toads often spend long periods inside these steep-sided hoofprints (Figure 4), despite repeated attempts to climb out (both in the absence or in the presence of ants: E. Cabrera-Guzmán unpublished data).

Especially at times and in places where alternative shelter-sites (notably soil cracks) are scarce or absent, the availability of sloping hoofprints may greatly enhance habitat quality for metamorph cane toads. Importantly, hoofprints are likely to be present around ponds with high anthropogenic disturbance – a consistent predictor of cane toad usage [46], [47]. The ability to exploit this abiotic buffering is part of a more general tactic in cane toads of all body sizes, whereby they seek out diurnal shelter-sites that reduce dehydration and thermal stress [36], [38]. The capacity to flexibly use such opportunities may be critical to invasion success, as the toads have encountered increasingly hotter and drier conditions over the course of their Australian invasion [60], [61].

Our field surveys showed that hoofprints differ substantially in density, depth and diameter. These parameters shift considerably within three metres' distance from the edge of the waterbody, plausibly reflecting soil moisture levels (which constrain the depth of penetration of bovine and equine hooves). Spatial variation in hoofprint densities and sizes likely also reflects the amount of time that ungulates spend in each area (i.e. they spend longer actually drinking than they do approaching or leaving), and the rate at which the steep sides of the hoofprints eventually collapse inwards. These variations undoubtedly modify thermal regimes and rates of desiccation, creating the same kinds of variation as occurs among the many microhabitat types used as diurnal shelters by adult toads [36]. Hoofprints vary not only in their depth and diameter [28], [30], but also in their contents. New footprints located very close to the edge of the pond often contain water, whereas those further from the waterbody often accumulate leaf-litter (E. Cabrera-Guzmán unpublished data).

Although hoofprints can enhance habitat quality for metamorph toads, the use of these refuges is constrained by other factors. Notably, quadrats containing hoofprints attracted more predatory ants than did open quadrats, likely reflecting the greater availability of prey (insects, anurans, etc.) inside depressions in the ground. Foraging meat ants prey on a wide variety of invertebrates, and also take carcasses [32]. As a consequence, toads stayed for shorter periods of time inside hoofprints if ants were present. In our trials, the arrival of ants often stimulated departure by metamorph toads; successful attacks on metamorphs show the advantage of retreat in this situation. The smallest toads often showed no overt avoidance of ants, even after the first contact (as noted by Ward-Fear et al. [27]). The end result of this interaction is to reduce the habitat quality of hoofprints for metamorph toads, inducing them to leave their current location in favour of an alternative refuge type, or another hoofprint that does not contain ants. Metamorph toads unfortunate enough to find themselves in a steep-sided hoofprint, where escape is difficult, may be at substantially greater risk from predatory ants (especially if toads are small and thus slow [62], or if they are not able to climb). The ants have more chances to repeatedly attack metamorphs in a steep-sided hoofprint; additionally the attachment forces and capacity of worker ants to carry objects that are many times their own mass [63], [64], [65] allow them to climb the vertical walls carrying a metamorph toad. Toads attacked and carried out of hoofprints by single worker ants sometimes struggled and escaped after they were removed from the hoofprint; but such toads may well have later died from their injuries (based on subsequent mortality rates of metamorph toads that escaped from ant attack: [32]). The social and cooperative behaviour of ants allow them to successfully prey on large and active prey [64], [66], thus more successful predation events occur when more meat ants participate in the attack and transport of toads (E. Cabrera-Guzmán unpublished data).

The different outcome for a metamorph toad spending time in a steep-sided hoofprint versus a gently-sloping hoofprint can thus offer management implications. For example, elevated troughs that reduce or eliminate damp muddy margins to watering-areas may (1) substantially reduce overall availability of hoofprints (by reducing the area of moist ground near a watering point), and (2) eliminate the hoofprint profiles that favour cane toads (because the trough is not surrounded by sloping ground) Thus, the construction of elevated water sources for stock could substantially decrease habitat quality for invasive cane toads in this system.

In conclusion, our work suggests two opposing effects of livestock grazing on pondside habitats from the perspective of a metamorph cane toad: the creation of numerous refuge-sites offering moist cool conditions otherwise unavailable in the surrounding landscape, and the creation of numerous “traps” in which toads may accumulate (due to steep sides preventing easy egress) and where they are more vulnerable to attacks and predation by meat ants, as well as to drowning. Livestock grazing covers >52% of the Australian continent [67], [68], and is the dominant form of land use over most of the toad's range in the Australian tropics. In combination with other landscape modifications wrought by grazing enterprises, such as the provision of additional watering points [56], [69], and the introduction of edible beetles for biocontrol of cowpats [57], [70], [71], the dramatic modification of waterside substrates through physical impacts of bovine and equine hooves may have contributed to the remarkable success of invasive cane toads in Australia.
